# Chondroitin polymerizing factor (CHPF) promotes development of malignant melanoma through regulation of CDK1

**DOI:** 10.1038/s41419-020-2526-9

**Published:** 2020-07-01

**Authors:** Wei Sun, Fang Zhao, Yu Xu, Kai Huang, Xianling Guo, Biqiang Zheng, Xin Liu, Zhiguo Luo, Yunyi Kong, Midie Xu, Dirk Schadendorf, Yong Chen

**Affiliations:** 1 Department of Musculoskeletal Oncology, Fudan University Shanghai Cancer Center, Fudan University, Shanghai, China; 2 Department of Oncology, Shanghai Medical College, Fudan University, shanghai, 200032 China; 3 Department of Dermatology, University Hospital Essen, Hufelandstrasse 55, 45122 Essen, Germany; 4 Brandon Reginal Hospital, HCA Healthcare/USF Morsani College of Medicine, Brandon, FL USA; 5 Department of Oncology, Dermatology Hospital, Tongji University, Shanghai, China; 6 Department of Oncology, Shanghai Tenth People’s Hospital, Tongji University, Shanghai, China; 7 Tongji University Cancer Center, Shanghai, 200072 PR China; 8 Department of Medical Oncology, Fudan University Shanghai Cancer Center, Fudan University, Shanghai, China; 9 Department of Pathology, Fudan University Shanghai Cancer Center, Fudan University, Shanghai, China

**Keywords:** Immunochemistry, Skin cancer

## Abstract

Chondroitin polymerizing factor (CHPF) is an important member of glycosyltransferases involved in the biosynthesis of chondroitin sulfate (CS). However, the relationship between CHPF and malignant melanoma (MM) is still unknown. In this study, it was demonstrated that CHPF was up-regulated in MM tissues compared with the adjacent normal skin tissues and its high expression was correlated with more advanced T stage. Further investigations indicated that the over-expression/knockdown of CHPF could promote/inhibit proliferation, colony formation and migration of MM cells, while inhibiting/promoting cell apoptosis. Moreover, knockdown of CHPF could also suppress tumorigenicity of MM cells in vivo. RNA-sequencing followed by Ingenuity pathway analysis (IPA) was performed for exploring downstream of CHPF and identified CDK1 as the potential target. Furthermore, our study revealed that knockdown of CDK1 could inhibit development of MM in vitro, and alleviate the CHPF over-expression induced promotion of MM. In conclusion, our study showed, as the first time, CHPF as a tumor promotor for MM, whose function was carried out probably through the regulation of CDK1.

## Introduction

Malignant melanoma (MM) is a highly malignant neoplasm originating from melanocytes in the basal layer of epidermis, which is predominant in the skin or mucosa adjacent to the skin^1^. Because MM has no specific markers for early clinical diagnostic and is characterized by rapid progression, satellite foci, local lymph node metastasis and distant metastasis could appear soon after the onset of melanoma, causing the approximate 10% 5-year survival rate for patients with advanced MM^2^. Although >90% of patients with early MM could be clinically cured through surgical resection treatment, advanced MM patients could not benefit from the surgery because of the rapid progression and metastasis of MM^3^. Following the proposal and rapid development of cancer immunotherapy^4^, several targeted immunotherapy drugs such as Ipilimumab^5^ and Nivolumab^6^ have been approved by FDA in clinical treatment of MM and proved to prolong survival of patients with advanced MM^7^. However, the prognosis of MM is still poor and far from satisfactory. It has been well documented that the over-expression of cancer-promoting genes plays an important role in the development and progression of human cancers^8^. Therefore, the exploration of cancer-promoting genes and the underlying mechanism of their promotion effect on MM not only could deepen the understanding of the molecular mechanism of MM, but also have important significance for clinical diagnosis and treatment.

Chondroitin sulfate (CS) is a polysaccharide which have essential functions in cell adhesion, cell division and neural network formation^9,10^. The biosynthesis of CS consists of several complex steps, in which the involvement of 6 glycosyltransferases has been identified^11^. As one of them, chondroitin polymerizing factor (CHPF), which is also known as chondroitin sulfate synthase-2 (CSS2)^12^, is a type II transmembrane protein consisted of 775 amino acids and has been proved to function mainly during the extension of CS biosynthesis^13^. Watanabe et al. reported that the CS chains in the cartilage of CHPF knockout mice has a shorter length compared with the wild-type mice^14^. Although the functional research of CHPF is still in the initial stage, its role in the development of some types of human cancers has been revealed^15,16^. For example, Fan^15^ et al. indicated that CHPF was upregulated in glioma and could promote the growth and inhibit cell apoptosis of glioma cells, suggesting its potentially oncogenic role. However, the relationship between CHPF and MM has never been investigated and remains unclear.

In this work, our studies revealed that CHPF acts as a tumor promoter for the development and progression of MM. The profound exploration of the mechanism underlying the promotion effect of CHFP on MM indicated that CHPF may promote MM through the regulation of CDK1. For the first time, this study clarifies the role of CHPF in MM, which may be identified as novel therapeutic target in treatment strategies for MM.

## Materials and methods

### Cell lines and cell culture

Human cutaneous melanoma cell line A375 was purchased from BeNa Technology (Hangzhou, Zhejiang,China). Human uveal melanoma cell line OM431 was supplied by SxBio Biotechnology Co., Ltd. (Shanghai, China). Cells were cultured in DMEM with 10% FBS. The incubator was maintained at 37 °C with 95% CO_2_ and humidity 70–80%.

### Reagents and antibodies

DMEM (Invitrogen, Carlsbad, CA, USA) with 10% FBS (Gibco, Rockville, MD, USA) was used for cell culture. 3-(4,5-dimethyl-2-thiazolyl)-2,5-diphenyl-2-H-tetrazolium bromide (MTT) (Sangon Biotech, Shanghai, China) was used for MTT assay.

The primary antibodies for Western blot were as follows: Anti-CHPF, anti-N-cadherin, anti-BRCA1, anti-CDK1, anti-BIRC5/Survivin, anti-Vimentin (Abcam, Cambridge, MA, USA), anti-Snail (CST, Danvers, MA, USA), anti-E2F1 (Tianjin Saierbio, Tianjin, China), anti-GAPDH (Bioworld, St. Loui, MN, USA). Secondary antibody HRP goat anti-rabbit IgG (Beyotime, Beijing, China). Secondary antibody HRP goat anti-mouse IgG and HRP goat anti-rabbit IgG (Beyotime, Beijing, China).

The Anti-CDK1 (Abcam, Cambridge, MA, USA) and anti-Ki-67 (Abcam) were used for immunohistochemistry (IHC).

### Plasmids and siRNA

The BR-V lentiviral vector system, pHelper 1.0 vector, pHelper 2.0 vector and 293T cells or TOP10 E. coli competent cells were used to produce lentiviruses. Human CHPF-targeting shRNA (targeting sequence: 5’-AGCTGGCCATGCTACTCTTTG-3’) and control insert sequence (5’-TTCTCCGAACGTGTCACGT-3’) were designed and cloned into lentiviral vector BR-V108. Transfection was performed in A375 and OM431 cells (8 × 10^6^ TU/mL). The human CDK1-targeting shRNA (5’-AGACTAGAAAGTGAAGAGGAA-3’) were designed and cloned into BR-V108 lentiviral vector and transfected into A375 cells (1 × 10^6^ TU/mL). The control shRNA lentivirus plasmids were purchased from Shanghai Bioscienceres (Shanghai, China), which also designed all the shRNAs. Moreover, full length of CHPF was cloned into lentivirus vector Ubi-MCS-3FLAG-CBh-gcGFP-IRES-puromycin (BR-V112) (Shanghai Bioscienceres, Shanghai, China) for constructing CHPF over-expressed cells.

### Immunohistochemistry (IHC)

Paraffin-embedded human melanoma tissue microarrays (TMA) ME2082c and ME2081 were purchased from Xi’an Alenabio (Xi’an, Shanxi, China). The appendant follow-up information was used in the statistical analysis between CHPF expression and tumor characteristics. The samples were collected from patients being completely informed. The study was approved by the Ethics Committee of Fudan University. The sections were deparaffinized, citrate antigen repaired, blocked by 3% H_2_O_2_ for 10 min at room temperature, and treated by 5% goat serum for 15 min at room temperature. Then the staining of the tissue sections by diluted primary anti-CHPF or anti-CDK1 was performed 4 °C overnight. After washing, the tissue sections were stained with DAB, and counterstained with hematoxylin, dehydrated with ethanol and mounted with resin. Images were captured using ImageScope v11 (Leica, Buffalo Grove, IL, USA). The CHPF or CDK1 expression levels in each spot on TMA was assessed by the intensity (1–4, from weak to strong) and the percentage of CHPF or CDK1 positive cells (0–100%, low to high).

### Western blot analysis

Total proteins from cells were extracted using ice-cold RIPA lysis buffer (Nanjing KeyGen Biotech, Nanjing, Jiangsu, China) and the concentration of proteins was determined by BCA protein reagent kit (Takara, Otsu, Japan). 10% SDS-PAGE gel was used to separate the proteins and then equal amount of proteins (20 μg) was transferred from the gel to poly(vinylidene fluoride) (PVDF) membrane. The membranes were blocked with 5% BSA for 1 h at room temperature and then incubated with primary antibodies including Anti-CHPF (1:1000), anti-N-cadherin (1:1000), anti-BRCA1 (1:1000), anti-CDK1 (1:2000), anti-BIRC5/Survivin (1:1000), anti-Vimentin (1:1000), anti-Snail (1:1000), anti-E2F1 (1:500), and anti-GAPDH (1:3000) at 4 °C overnight. After washing, the membranes were incubated with secondary antibodies. The expression of proteins was visualized by ECL-Plus™ Western blotting system, and proteins were detected with an X-ray imaging analyzer (Kodak, Rochester, NY, USA). GAPDH was used as the internal standard.

### Quantitative RT-PCR

TRIzol^®^ reagent (Thermo Fisher Scientific, Waltham, MA, USA) was used for the extraction of total RNA from each sample according to the manufacturer’s protocol. HiScript Q RT SuperMix for qPCR (+gDNA wiper) (Vazyme, Nanjing, Jiangsu, China) was utilized for generating cDNA according to the manufacturer’s protocol. Then the qRT-PCR was performed by using SYBRVR Premix Ex TaqTM (Takara, Otsu, Japan) and Mx3000P QPCR System (Agilent, Santa Clara, CA, USA). GADPH was used as an endogenous reference. The primers used in qRT-PCR were as followed:

CHPF-F: 5’-GGAACGCACGTACCAGGAG-3’

CHPF-R: 5’-CGGGATGGTGCTGGAATACC-3’

CDK1-F: 5’-TCAGGATGTGCTTATGCAGGATTC-3’

CDK1-R: 5’-TCCATGTACTGACCAGGAGGGA-3’

GAPDH-F: 5’-TGACTTCAACAGCGACACCCA-3’

GAPDH-R: 5’-CACCCTGTTGCTGTAGCCAAA-3’.

### MTT assay

Cells in exponential growth phase were trypsinize and then were seeded onto a 96-well plate (2000 cells/well). Twenty microliter of MTT (5 mg/mL) solution (Genview Scientific, Inc.) was added to the cells 4 h prior to the culture termination. Absorbance values at 490 nm were measured by microplate reader (Tecan Group, Ltd; M2009PR) for each well after 1, 2, 3, 4, 5 days of growth, and the reference wavelength was 570 nm. The absorbance could represent vital cell percentage, and the cell viability ratio was calculated according to the equation: Cell viability (%) = optical density (OD) treated/OD control × 100%.

### Celigo image cytometer

A375 cells were collected after the transfection of CHPF (for over-expression), shCDK1, CHPF + shCDK1 and the corresponding negative controls. Then cells were seeded into 96-well plates with a cell density of 2000 cells per well. Cells were further cultured in an incubator with 5% CO_2_ at 37 °C for 24 h. Celigo image cytometer (Nexcelom Bioscience, Lawrence, MA, USA) was utilized to take the cell images at day 1, 2, 3, 4, 5. Cell counting was also accomplished by Celigo image cytometer and the cell proliferation curve was drawn.

### Colony formation assay

Cells in logarithmic phase were digested by trypsin, resuspended, counted and seeded in 6-well plates at cell density of 400–1000 cells/well. Cells were incubated for 14 days to form colonies. Then cells were washed three times by PBS, fixed by paraformaldehyde for 1 h, stained with giemsa for 20 min, washed three times and then photographed with a digital camera. The number of colonies (>50 cells/colony) was counted under microscopy (MicroPublisher 3.3 RTV; Olympus, Tokyo, Japan). All assays were performed in triplicate.

### Flow cytometry

At 48 h after transfection, cells were collected, washed and centrifuged, with the aspiration of supernatant. Then the cells were digested with trypsin and resuspended in the same medium. Then the cells were stained with Annexin V-allophycocyanin (APC, 10 μL) for 15 min in the dark at room temperature. Annexin-V Allophycocyanin/Propidium Iodide Kit (eBioscience; Thermo Fisher Scientific, Inc.) was used as the tool for analyzing the percentage of apoptotic cells in each group.

### Human apoptosis antibodies array

The differentially expressed apoptosis-related proteins induced by CHPF knockdown in A375 cells were detected by Human apoptosis antibody array (RayBio, Norcross, GA, USA). Human apoptosis antibody array-membrane was placed into a dish and cell lysates were added to each well for incubation at 4 °C with gentle shaking overnight. The membranes were washed and then incubated with lyophilized biotinylated antibodies for 1 h on a rocking platform shaker. Then the excess molecules were washed away and the membranes were further incubated with horse radish peroxidase-conjugated streptavidin for 30 min. The expression levels of proteins were analyzed using Gelpro Analyzer software (Media Cybernetics, Rockville, MD, USA).

### Migration assays

Wound-healing assay was performed to evaluate cell migration ability of A375 cells transfected with shCtrl or shCHPF. The A375 cells were cultured till the cell density 90% was obtained, then a line wound was scratched by scraping a 100 μL tips across the confluent cell layer. The cells were washed by PBS for three times to remove the cellular debris. Then the cells were cultured with serum-free medium 24 h at 37 °C. The photos of the wounds were acquired by a light microscope (DFC500; Leica) at 0, 8, and 24 h after scratching, and the relative edges of cells were assessed.

Transwell assay was also used for detecting cell migration, which was operated by polycarbonate membrane corning transfer experiment kit (Corning) according to the manufacture’s protocols. Briefly, the transfected A375 and OM431 cells cultured by serum-free DMEM medium were loaded into the upper chamber. DMEM medium supplemented with 30% FBS was added to the lower chamber as the chemoattractant. After 24 h of incubation, the cells remaining in the upper chamber were removed. The numbers of migrated cells, as well as the cells in the lower chamber, were estimated in 5 randomly selected views using microscopic inspection (Olympus) after the staining of giemsa for 20 min.

### RNA-sequencing

The RNA-sequencing analysis was performed by Genechem (Shanghai, China). Briefly, total RNA of the cell samples (A375 transfected with shCtrl or shCHPF, 3v 3) was extracted using TRIzol reagent (Thermo Fisher Scientific, Waltham, MA, USA) according to manufacturer’s protocols. The purified RNA was quantified by using a NanoDrop 2000 (Thremo Fisher Scientific, Waltham, MA, USA). The libraries for RNA-sequencing were constructed by the TruSeq Stranded mRNA LT Sample Prep Kit (Illumina, San Diego, CA, USA) according to the manufacturer’s instructions and then scanned by Affymetrix Scanner 3000 (Affymetrix, Santa Clara, CA, USA). The differentially expressed genes (DEGs) between the two groups were identified based on the threshold of absolute fold change >2 and FDR < 0.05. IPA (Qiagen, Hilden, Germany) was performed based on all the DEGs for analyzing the enriched functional annotations. The absolute value of the *Z* score greater than 2 is considered meaningful.

### Animal studies

All animal experiments performed here were approved by the Institutional Animal Care and Use Committee of Fudan University. Six-week-old male BALB/c nude mice were purchased from Shanghai SLAC Laboratory Animal Co. Ltd (Shanghai, China), housed at pathogen-free condition, and divided into two groups (shCtrl Group and shCHPF Group) randomly before experiments. A375 cells (4 × 10^6^) suspended with PBS were subcutaneously injected into mice through right axilla for the construction of mice xenograft model. The mice were cultured for further 30 days post injection and the collection of data began from 5 days post injection. The volume of tumors was estimated based on the measurement of length and width at 5, 7, 11, 14, 20, 23, 27, 30 days post injection. Finally, mice were sacrificed through injection of pentobarbital sodium, and the tumors were removed for taking photos and weighting.

### Statistical analysis

Statistical analyses were performed using SPSS 20.0 (SPSS, Chicago, IL, USA) and GraphPad prism 6.0 (GraphPad, La Jolla, CA, USA). Student’s *t*-Test and Chi-square test were used during the data analysis. 2^−∆∆Ct^ method were used during the qPCR assays. Data are presented as the mean ± SD (*n* ≥ 3). *P* < 0.05 was considered as statistically significant and the absolute value of the Z score greater than 2 is considered meaningful in IPA.

## Results

### Identification of CHPF as a potential tumor promoter in MM

**Fig. 1:**
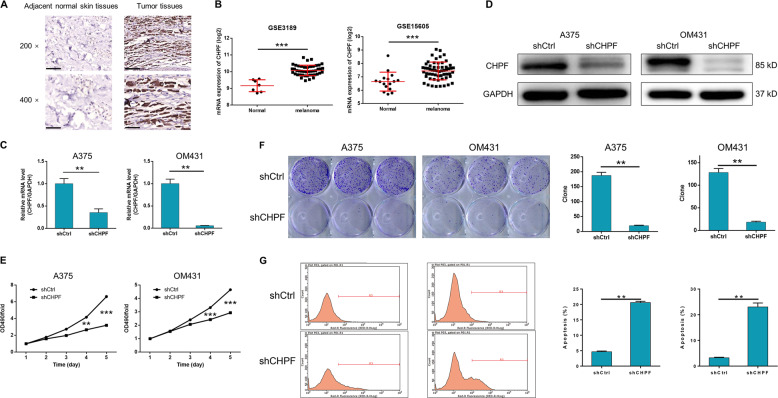
Role of CHPF in MM tissues and cells. **a** The expression of CHPF in MM tissues and adjacent normal skin tissues were detected by IHC analysis (Magnification ×400). **b** The upregulated expression of CHPF in MM tissues was proved through data mining of GSE3189 and GSE15605 datasets. **c**, **d** The efficiencies of CHPF knockdown in A375 and OM431 cells were detected by qPCR and Western blot analysis, respectively. **e** MTT assay was performed to detect the cell proliferation rate of A375 and OM431 cells transfected with shCtrl or shCHPF. **f** Colony formation assay was utilized to compare the colony formation ability of MM cells transfected with shCtrl or shCHPF. **g** Flow cytometry analysis was performed to show the apoptotic cell percentage in A375 and OM431 cells with or without CHPF knockdown. The data were expressed as mean ± SD (*n* ≥ 3), *P < 0.05, **P < 0.01, ***P < 0.001.

In order to investigate the role of CHPF, thus exploring the molecular mechanism of MM, the expression level of CHPF was detected by immunohistochemistry (IHC) analysis of a human MM tissue microarray (TMA). In total, 184 MM tissues and 16 adjacent normal skin tissues were included and the detection results indicated that the expression of CHPF was significantly upregulated in MM tissues as illustrated by the representative images in Fig. 1a and statistical analysis in Table 1. Moreover, the expression profiling of dataset GSE3189 (7 normal tissues vs. 45 MM tissues) and GSE15605 (16 normal tissues vs. 58 MM tissues) also proved that CHPF was over-expressed in MM tissues compared with normal tissues (Fig. 1b). Furthermore, statistical analysis was performed to analyze the relationship between CHPF expression and tumor characteristics of MM patients, which showed that high CHPF expression was positively related with more advanced T stage (Table 2 and 3), with a Pearson correlation coefficient of 0.226 (*P* < 0.05) (Table S1). All the above results demonstrated the potential of CHPF as a tumor promoter in MM.

**Table 1:** Expression patterns in MM tissues and adjacent normal skin tissues revealed in immunohistochemistry analysis.

CHPF expression	Tumor tissues		Adjacent normal skin tissues		*p* value
	Cases	Percentage	Cases	Percentage	
Low	119	64.7%	16	100%	<0.001***
High	65	35.3%	0	–

**Table 2:** Relationship between CHPF expression and tumor characteristics in patients with malignant melanoma.

Features	No. of patients	CHPF expression		*p* value
		low	high	
All patients	86	38	48	
Age (years)				0.360
<51	41	16	25	
≥51	45	22	23	
Gender				0.629
Male	41	17	24	
Female	45	21	24	
T infiltrate				0.037*
T1	6	4	2	
T2	16	11	5	
T3	3	0	3	
T4	61	23	38	
lymphatic metastasis (N)				0.941
N0	79	35	44	
N1	7	3	4	
Stage				0.097
I	12	9	3	
II	65	25	40	
III	8	4	4	
IV	1	0	1

**Table 3:** Relationship between CHPF expression and tumor characteristics in patients with malignant melanoma.

		CHPF
T Infiltrate	Pearson correlation coefficient	0.226
	Significance (two-tailed)	0.036*
	N	86

### Knockdown of CHPF inhibits the development and progression of MM

**Fig. 2:**
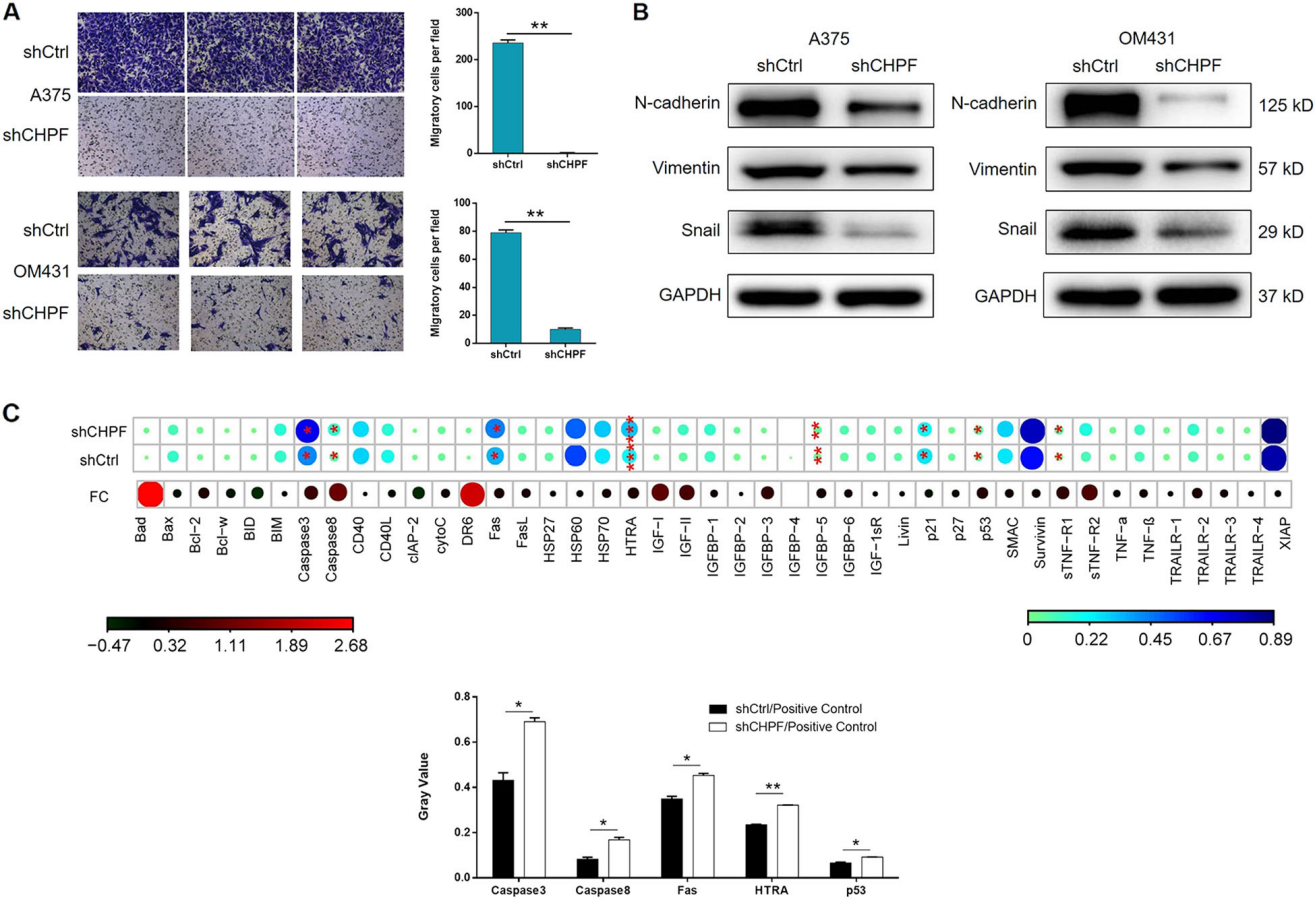
The regulation effect of CHPF on cell migration and EMT/apoptosis related proteins. **a** Transwell assay was performed to compare the cell migration ability of MM cells treated with shCtrl or shCHPF. **b** The expression of EMT biomarkers including N-cadherin, Snail and Vimentin were detected by Western blot analysis in MM cells treated with shCtrl or shCHPF. **c** Human apoptosis antibody array was utilized to illustrate the regulation of the expression of apoptosis related proteins by CHPF knockdown. The data were expressed as mean ± SD (*n* ≥ 3), *P < 0.05, **P < 0.01, ***P < 0.001.

**Fig. 3:**
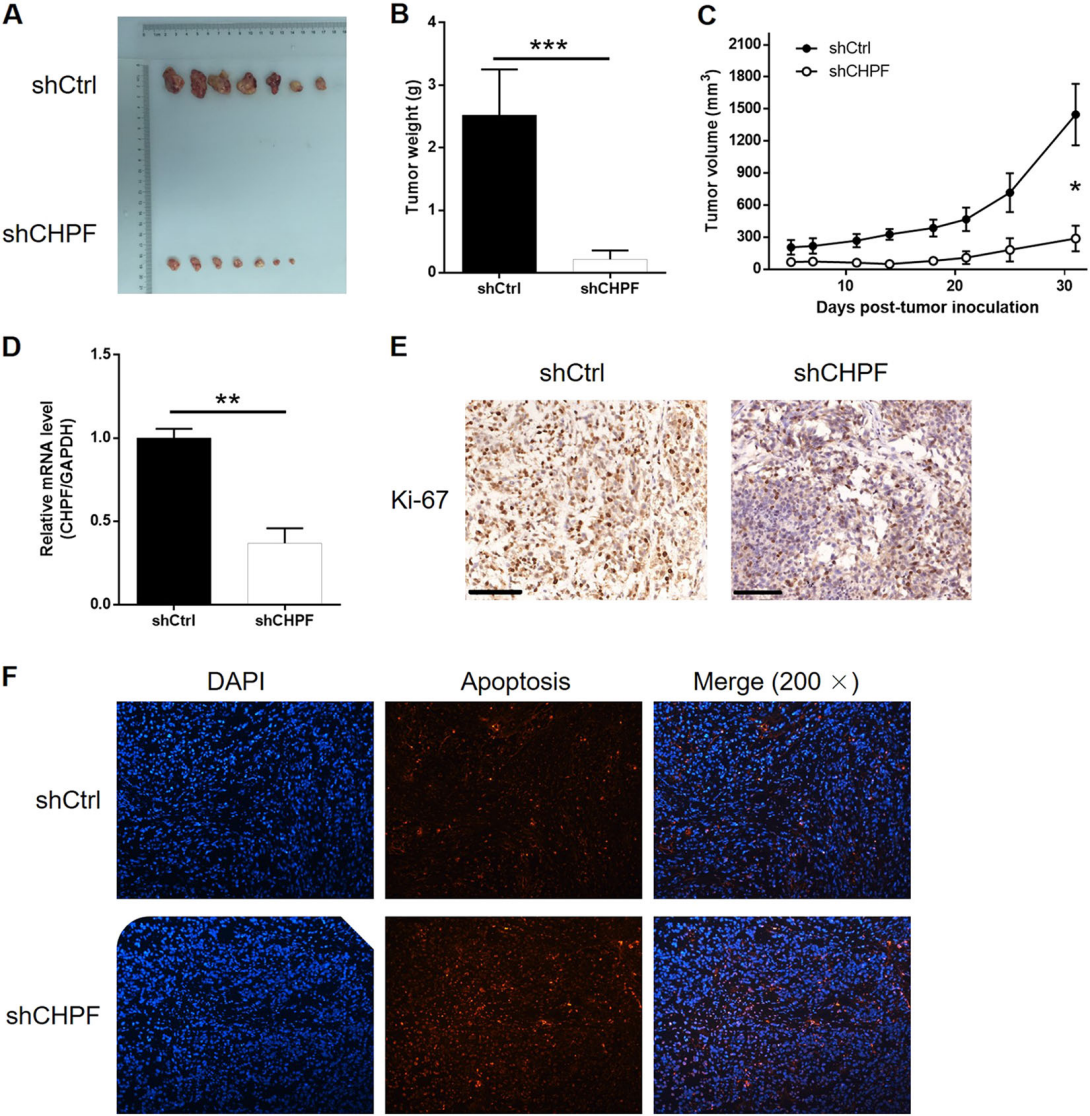
The knockdown of CHPF inhibits MM tumor growth in vivo. **a** The photo of xenograft tumors removed from the mice models in shCtrl and shCHPF groups. **b** The weight of tumors was measured after the sacrifice of mice models. **c** The volume of tumors was measured and calculated during experiments. **d** The mRNA expression of CHPF in tumors was detected by qPCR. **e** The Ki-67 index of the removed tumors was evaluated by IHC analysis (Magnification ×100). **f** The Tunel staining of the sectioned tumors was performed to assess the apoptosis rate. The data were expressed as mean ± SD (*n* ≥ 3), *P < 0.05, **P < 0.01, ***P < 0.001.

For further exploring the effects of CHPF on development and progression of MM, human cutaneous melanoma cell line A375 and human uveal melanoma cell line OM431 were selected for the construction of cell model. The cells were transfected with lentivirus expressing short hairpin RNA (shRNA) designed specifically for silencing CHPF as shCHPF group or scrambled lentivirus as negative control (shCtrl). The green fluorescence, resulted from the GFP tag on the lentivirus vector, detected inside the cells was used to represent the successful transfection, which proved >80% transfection efficiencies of both cell lines (Fig. S1A). qPCR and Western blot analysis (WB) were performed to evaluate the knockdown efficiency and suggested a >50% knockdown of CHPF for both cell lines (Fig. 1c, d). Subsequently, the effects of CHPF knockdown on cell proliferation, colony formation, cell apoptosis and cell migration were estimated by MTT assay (Fig. 1e), colony formation assay (Fig. 1f), flow cytometry (Fig. 1g) and Transwell assay (Fig. 2a), respectively. The results indicated that the knockdown of CHPF significantly inhibited the proliferation rate (>40% inhibition, *P* < 0.01), colony formation ability (>80% inhibition, *P* < 0.01) and cell migration ability (>80% inhibition, *P* < 0.01), while significantly promoting the cell apoptosis of both A375 and OM431 cells (>4-fold promotion, *P* < 0.01). Otherwise, expression levels of several epithelial-mesenchymal transition (EMT) biomarkers such as N-cadherin, Snail and Vimentin were also detected by WB and their downregulated expression in shCHPF group were in consistence with the results of Transwell assay (Fig. 2b). Moreover, the differentially upregulated apoptosis related proteins including Caspase3, Caspase8, Fas, HTRA, and p53 identified by the detection of human apoptosis antibody array (shCHPF vs. shCtrl) suggested the potential involvement of Fas and p53 signaling pathway in the regulation ability of CHPF on MM (Fig. 2c). For the sake of further verifying the role of CHPF in MM, mice xenograft models were constructed by subcutaneous injection of A375 cells with or without CHPF knockdown. The observation of the removed solid tumors, as shown in Fig. 3a, demonstrated the significantly suppressed tumor growth of MM in mice of shCHPF group compared with that of shCtrl group, which was also proved by the lighter tumor weight (Fig. 3b) and smaller tumor volume (Fig. 3c). In addition, following the verification of CHPF expression in xenograft tumors by qPCR (Fig. 3d), Ki-67 index was also detected by IHC analysis, which showed a significant downregulation in shCHPF group and was in line with the inhibited tumor growth (Fig. 3e). Collectively, all the above results clarified that CHPF knockdown could inhibit the development and progression of MM, which was consistent with its clinical relevance.

### **T**he exploration of downstream mechanism underlying the regulation of MM by CHPF

**Fig. 4:**
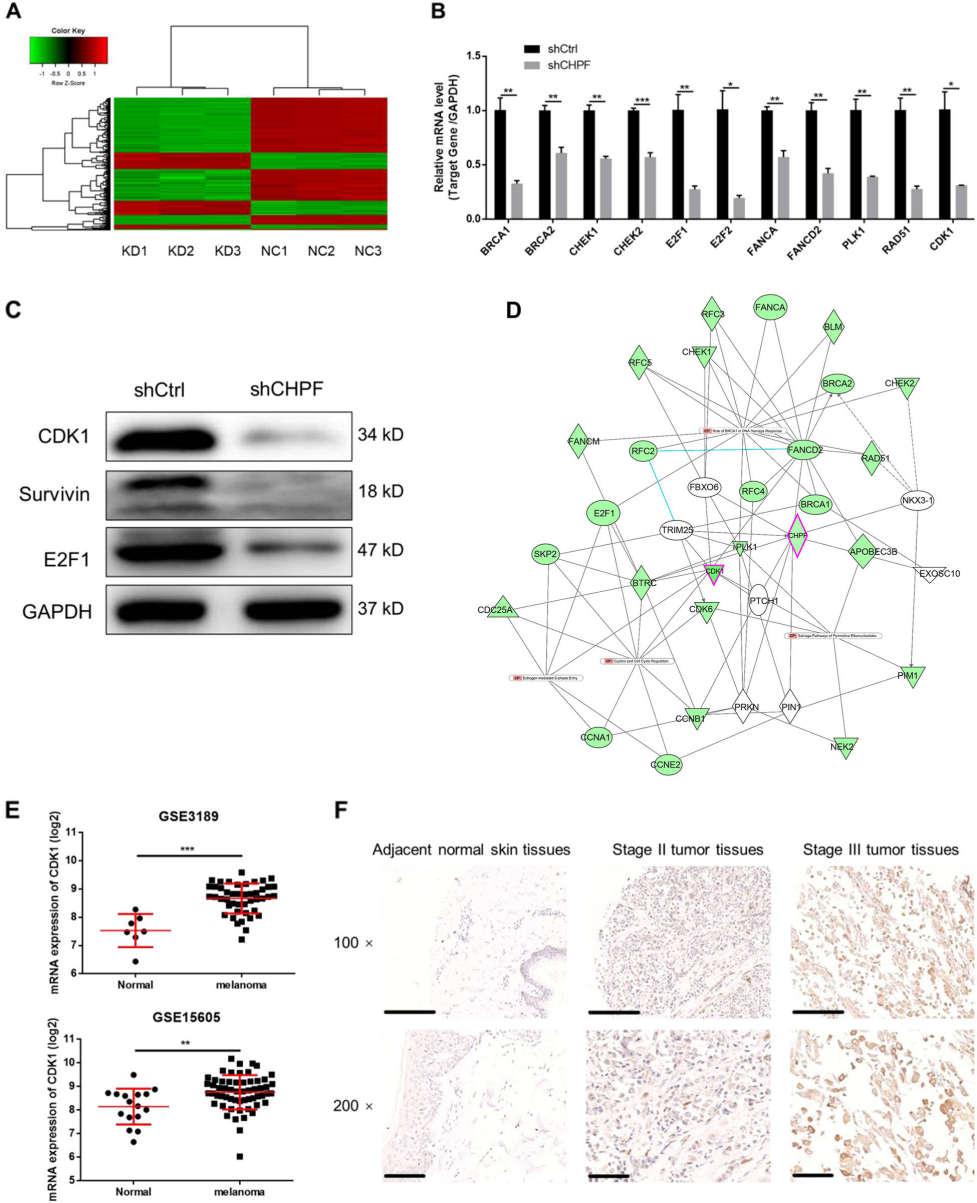
Exploration of underlying mechanism by RNA-sequencing and IPA analysis. **a** The heatmap of DEGs identified by RNA-sequencing of cells treated with shCtrl (*n* = 3) or shCHPF (*n* = 3). **b** IPA analysis of canonical signaling pathway was performed to identify the pathways regulated by CHPF knockdown. The dots were scaled by –Log (*p* value). The pathways in red were activated, and these in blue were inhibited. **c** The expression of several most significantly differentially expressed genes identified by RNA-sequencing, including BRCA1, CDK1, Survivin, and E2F1 was further detected in OM431 cells. **d** The IPA analysis of CHPF-related interaction network. **e** The upregulated expression of CDK1 in MM tissues was proved by data mining of GSE3189 and GSE15605 datasets. **f** The expression of CDK1 in normal skin tissue and stage II/III tumor tissues was detected by IHC analysis (Magnification 200×). *P < 0.05, **P < 0.01, ***P < 0.001.

In order to further elucidate the downstream mechanism underlying the inhibition of MM by CHPF knockdown, a transcriptional profiling was performed by RNA-sequencing based on A375 cells transfected with shCHPF (*n* = 3, as experimental group) or shCtrl (*n* = 3, as control group). In general, 689 differentially expressed genes (DEGs) were identified, among which there was 184 upregulated genes and 505 downregulated genes, based on the threshold of absolute fold change >2 and FDR < 0.05 (Fig. 4a, S1B, C). Ingenuity pathway analysis (IPA) of canonical signaling pathway and disease and function was subsequently performed to identify the CHPF knockdown induced enrichment of genes. The canonical signaling pathway analysis showed that several cell cycle related pathways including G2/M DNA damage check-point regulation, G1/S check-point regulation and cyclin and cell cycle regulation were affected by CHPF knockdown (Fig. 4b). Otherwise, it was noteworthy that cell cycle was also identified as the most significantly influenced function through the IPA disease and function analysis (Fig. S1D). 4 most significantly downregulated candidates including BRCA1, CDK1, Survivin, and E2F1 were then selected for WB verification in OM431 cells (Fig. 4c). Further combining with the CHPF-associated interaction network analysis by IPA (Fig. 4d), CDK1, which plays well-known important role in cell cycle and is the most significantly downregulated one in cells with CHPF knockdown, was supposed to be the potential downstream of CHPF in the regulation of MM. The expression profiling data collected from dataset GSE3189 and GSE15605 also proved the upregulated expression of CDK1 in MM tissues compared with normal tissues (Fig. 4e). Accordingly, the protein expression of CDK1 detected by IHC in TMA verified the upregulated expression of CDK1 in tumor tissues compared with normal skin tissues, which was similar with the manner of CHPF (Fig. 4f).

### Knockdown of CDK1 alleviated the promotion effect of CHPF over-expression on MM

**Fig. 5:**
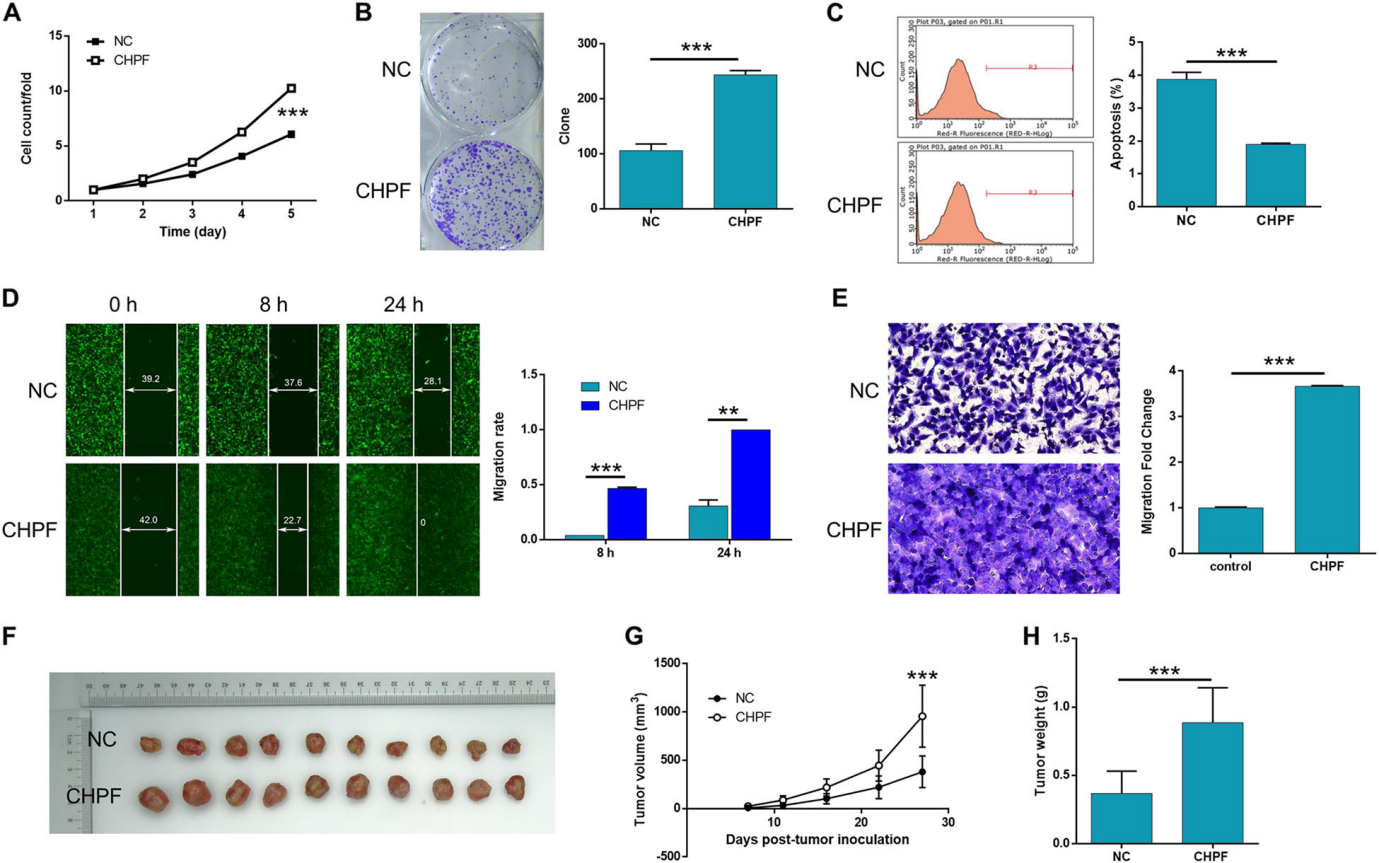
The effects of CHPF over-expression and CDK1 knockdown on MM. The effects of CHPF over-expression on proliferation, colony formation and apoptosis of A375 cells were examined by Celigo cell counting assay (**a**), colony formation assay (**b**) and flow cytometry assay (**c**), respectively. The effect of CHPF over-expression on cell migration of A375 cells was detected by wound-healing assay (**d**) and Transwell assay (**e**). (**f**–**h**) The promotion of tumor growth by CHPF over-expression was verified in mice xenograft model. The represented images were selected from at least 3 independent experiments. The data were expressed as mean ± SD (*n* ≥ 3), *P < 0.05, **P < 0.01, ***P < 0.001.

**Fig. 6:**
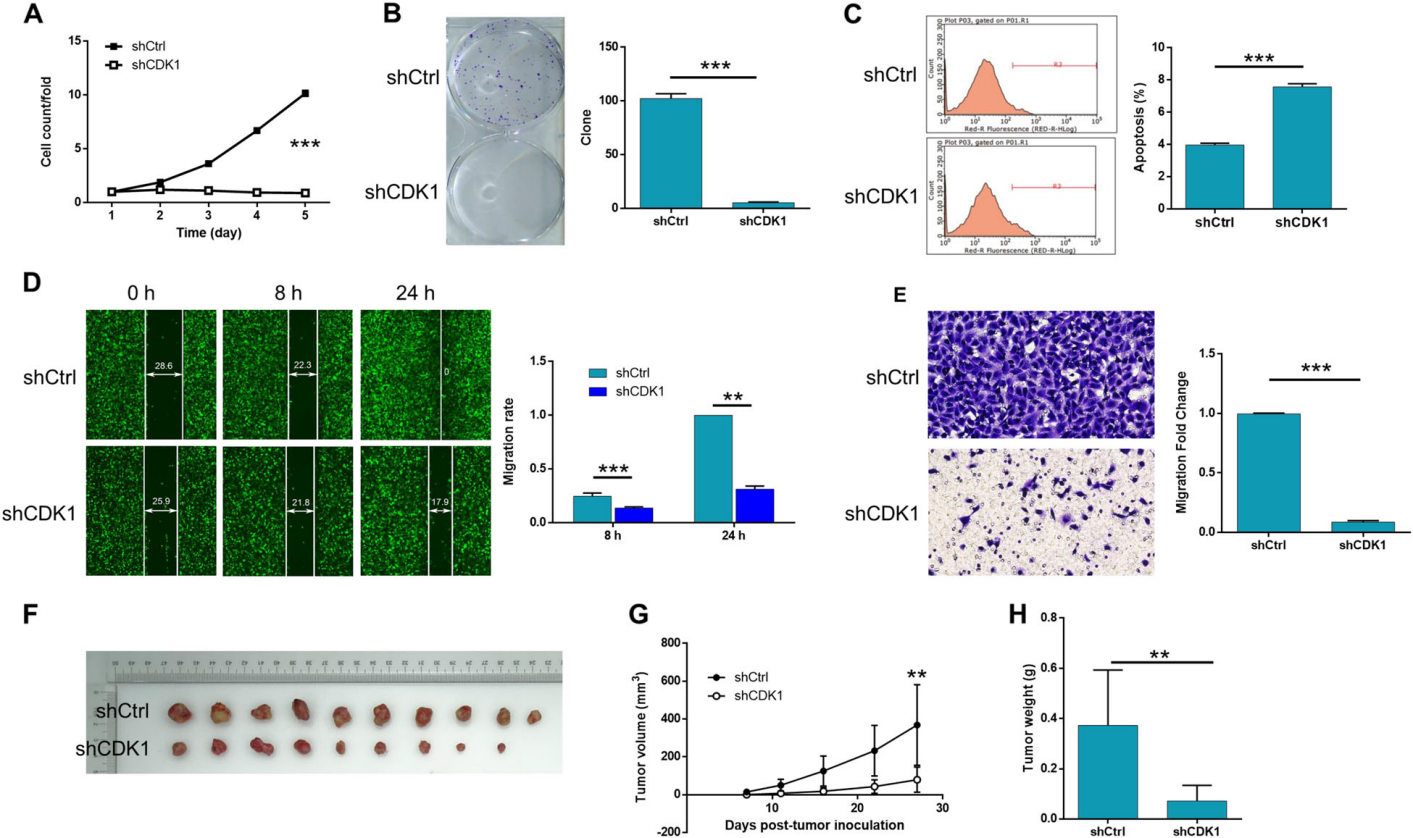
The effects of CDK1 knockdown on MM in vitro and in vivo. The effects of CDK1 knockdown on proliferation, colony formation and apoptosis of A375 cells were examined by Celigo cell counting assay (**a**), colony formation assay (**b**) and flow cytometry assay (**c**), respectively. The effect of CDK1 knockdown on cell migration of A375 cells was detected by wound-healing assay (**d**) and transwell assay (**e**). **f**–**h** The promotion of tumor growth by CDK1 knockdown was verified in mice xenograft model. The represented images were selected from at least 3 independent experiments. The data were expressed as mean ± SD (n ≥ 3), *P < 0.05, **P < 0.01, ***P < 0.001.

In order to verify that CHPF executes its regulation effects on MM through CDK1, the synergistic effects of them on the functions of MM cells were examined. The background expression of CHPF and CDK1 was tested in A375 cells and indicated that, although high expression levels were observed for both CHPF and CDK1, CDK1 exhibited relatively higher expression than CHPF (Fig. S2A). Therefore, A375 cells with CHPF over-expression (stable transfection, CHPF group), CDK1 knockdown (shCDK1 group) and simultaneous CHPF over-expression and CDK1 knockdown (OE + KD group) were constructed. The efficiencies of transfection, over-expression and knockdown were evaluated by fluorescence imaging, qPCR and WB as mentioned above (Fig. S2B–G). Herein, it was demonstrated that, compared with the negative control, over-expression of CHPF could significantly promoted the cell proliferation (>50% promotion, *P* < 0.001, Fig. 5a), colony formation (>2-fold promotion, *P* < 0.001, Fig. 5b) and cell migration ability (>3-fold promotion, *P* < 0.01, Fig. 5d, e) and inhibited cell apoptosis of MM cells (>50% inhibition, *P* < 0.001, Fig. 5c), which was in accordance with the inhibition effect of CHPF knockdown. Moreover, as indicated by cell experiments, CDK1 knockdown exhibited similar suppression effects on the development and progression of MM with CHPF (Fig. 5f–j). Most importantly, we observed that the knockdown of CDK1 in CHPF over-expressed cells could recover the expression of CHPF (Fig. 6a, b), and alleviate even reverse the promoted cell proliferation (Fig. 6c), colony formation (Fig. 6d), cell migration ability (Fig. 6e wound healing and Fig. 6f Transwell assay) and the inhibited cell apoptosis (Fig. 6g) by over-expression of CHPF. Therefore, the recovery experiments proved that the promotion effect of CHPF on the development and progression of MM may be mediated by CDK1.

## Discussion

**Fig. 7:**
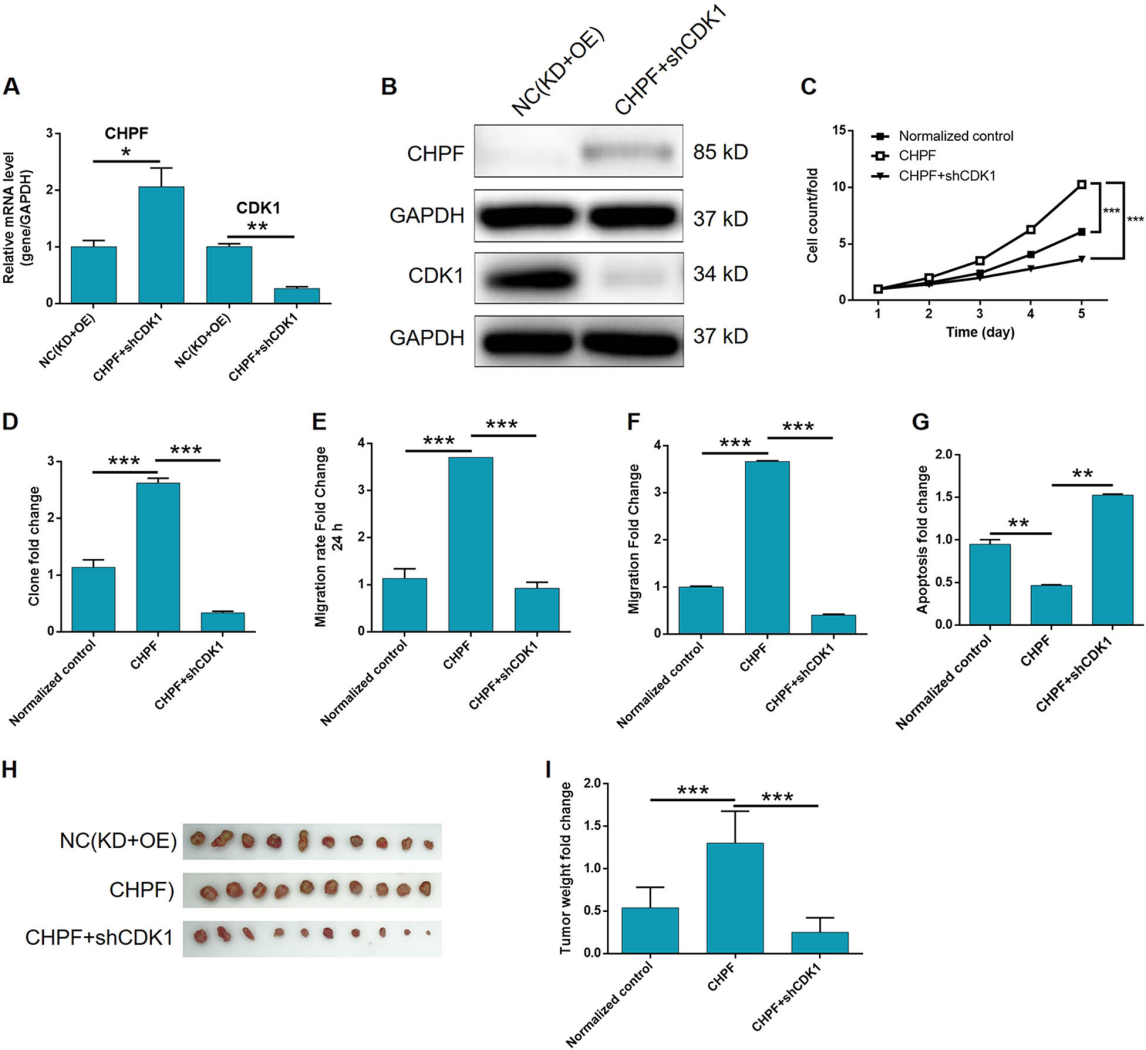
CDK1 knockdown alleviates the effects of CHPF over-expression on MM cells. The expression levels of CHPF and CDK1 in A375 cells with simultaneously CHPF over-expression and CDK1 knockdown were detected by qPCR (**a**) and WB (**b**), respectively. The influence of CDK1 knockdown on the CHPF over-expression induced changes of proliferation, colony formation, migration and apoptosis of A375 cells was detected by Celigo cell counting assay (**c**), colony formation assay (**d**), wound-healing assay (**e**), Transwell assay (**f**) and flow cytometry (**g**), respectively. **h**, **i** The in vivo experiments were also performed to reveal the influence of CDK1 knockdown on the CHPF over-expression induced promotion of tumor growth. All data were collected from at least three independent experiments and were normalized to corresponding negative control for facilitating comparison. The figures are representative data from at least three independent experiments. The data were expressed as mean ± SD (n ≥ 3), *P < 0.05, **P < 0.01, ***P < 0.001.

Malignant melanoma (MM) is the most aggressive malignant skin cancer, and it has been identified as the major cause of skin cancer related death worldwide^17^. Although MM in early stage could be effectively treated by surgical resection, the prognosis of MM is still poor because of its rapid progression, metastasis and the lack of efficient anti-MM drugs^3^. Therefore, the exploration of molecular mechanism and screening of therapeutic targets of MM have attracted more and more attention in the past decades. Till now, a lot of genes involved in the pathogenesis of MM have been revealed such as MITF^18^, CPEB4^19^, BRAF^20^, Flot2^21^, and KIT^22^, and some corresponding targeted drugs, such as vemurafenib^23,24^ (BRAF inhibitor) and imatinib mesylate^25^ (KIT inhibitor) have been developed for the treatment of melanoma. However, since the prognosis of MM is still poor and far from satisfactory, the deepening research of its molecular mechanism which could benefit for the discovery of more specific therapeutic targets for MM is still in urgent need. Recently, Luo et al. reported that CXCR7 is capable of promoting melanoma tumorigenesis by promoting Src-mediated eIF4E phosphorylation^26^. Bekeschus et al. reported the SLC22A16-mediated MM cell death induced by the combination of chemotherapy and physical plasma elicits^27^. In this study, the role of CHPF in the development and progression of MM was investigated for the first time (Fig. 7).

As one of the six glycosyltransferases involved in the biosynthesis of CS, CHPF (also known as CSS2) has been proved to have important functions in not only the biosynthesis of CS, but also the regulation of some types of malignant tumors. Wu et al. reported the high expression of CHPF in lung adenocarcinoma, and the inhibition of cell proliferation, as well as the promotion of cell apoptosis by knockdown of CHPF in vitro and in vivo which is probably through the regulation of MAPK signaling pathway^28^. They also indicated the promotion effect of CHPF in the development of glioma through investigating cell functions of U251 cells^15^. Otherwise, the relationship between the high expression of CHPF and tumorigenesis or development of colorectal cancer^29^ and head and neck squamous cell carcinoma^30^ has also been demonstrated. However, till now, the involvement of CHPF in MM was rarely reported and remains unclear.

In this study, the upregulated expression of CHPF in MM was proved by the IHC analysis of TMA including 184 MM tissues and 16 normal skin tissues. The expression profiling data collected from 2 sets of GSE datasets (GSE3189^31^ and GSE15605^32^) also supported the comparatively high expression of CHPF in MM. Through the construction of CHPF knockdown cell models based on A375 and OM431 cell lines and CHPF over-expression cell model based on A375 cell line, the effects of endogenic CHPF on the development and progression of MM were investigated in vitro. In line with the potential tumor promotion effect of CHPF indicated by the detection of tissues, the over-expression of CHPF promoted the cell proliferation and colony formation ability and simultaneously inhibited cell apoptosis. Accordingly, the knockdown of CHPF exhibited conversed effects which inhibited the cell and colony growth and promoted cell apoptosis. Moreover, the suppression of MM tumor growth by CHPF knockdown was further illustrated by mice xenograft model in vivo.

As mentioned before, one of the major obstacles in the improvement of MM prognosis is its trend to happen distant metastasis. Therefore, the influence of CHPF on the MM cell migration was also studied. The results indicated that the over-expression of CHPF could significantly promote cell migration ability of MM cells, while CHPF knock exhibited opposite effect. Furthermore, it has been well known and acknowledged that EMT plays critical role in the progression and metastasis of MM^33–35^. For example, Schittek et al. demonstrated that the expression and phosphorylation of YB-1 regulate tumorigenicity and invasiveness of melanoma by influencing EMT^36^. Herein, expression levels of several EMT-related biomarkers were also examined and showed significantly downregulation in MM cells with CHPF knockdown, indicative of the suppression of EMT by CHPF knockdown.

In addition, genome-wide expression profiling followed by Ingenuity Pathway Analysis (IPA) was performed for further exploring downstream mechanism underlying the regulation of CHPF on MM. Based on the DEGs identified by RNA-sequencing, the enrichment of genes affected by CHPF knockdown in canonical signaling pathway and IPA disease & function was analyzed and showed the high relevance of cell cycle. CDK1, which is tightly related to cell cycle, was not only the most significantly downregulated DEG in shCHPF group but also proved to be significantly downregulated in OM431 cells in shCHPF group. Moreover, the analysis of CHPF associated interaction network by IPA also indicated CDK1 as a potential downstream regulator of CHPF.

CDKs are a class of important serine/threonine protein kinases, which are activated after binding to cyclin and can catalyze the phosphorylation of substrates at different stages of mitosis, complete DNA synthesis and mitosis, thus causing cell growth and proliferation^37–39^. Positive feedback activation of CDK1 is the key event to initiate mitosis^40^. Increasing activity of CDK1-cyclinB1 kinase activates MPF, which is the key to coordinate the continuation of mitosis^41^. Except for its functions in cell cycle, CDK1 was also found to be abnormally expressed and mediate the regulation in various types of malignant tumors^42,43^. For example, the study of Saatci et al. showed that the T-DM1 resistance of HER2-positive breast cancer patients may be overcome by targeting Polo-like kinase 1 (PLK1) through the regulation of CDK1-dependent phosphorylation^44^. Moreover, CDK1 was also reported to be implicated in MM^45^. The work of Fujita et al. suggested CDK1 as a regulator of Sox2 and identified CDK1-Sox2 interaction as a potential therapeutic target in human melanoma^46^. In this study, the inhibition effect of CDK1 knockdown on MM, which is similar with CHPF, was also elucidated through the detection of cell proliferation, colony formation, cell apoptosis, and cell migration. More importantly, the promotion of MM by over-expression of CHPF could be impaired to some extent by simultaneous knockdown of CDK1, suggesting that CHPF may regulate the development and progression of MM through influencing CDK1.

In conclusion, the experimental data in this study identified that CHPF was upregulated in MM tissues and positively related with more advanced T stage. In vitro and in vivo detection demonstrated that CHPF acts as a tumor promoter in MM, probably through the regulation of CDK1. As far as we known, this is the first report concerning the role of CHPF in the development and progression of MM. However, more detailed mechanism such as the downstream signaling pathway involved in the regulation of MM by CHPF is still needed to be further explored.

## Supplementary information

Legends for supplementary figures

Figure S1

Figure S2

Figure S3
