# Supplementary material for: Chondroitin polymerizing factor (CHPF) promotes development of malignant melanoma through regulation of CDK1
Source: Cell Death Dis. 2020 Jul 1;11(7):496. doi: 10.1038/s41419-020-2526-9 (PMC7329816; doi:10.1038/s41419-020-2526-9)
Supplement: Supplementary file 1 — Legends for supplementary figures [file 41419_2020_2526_MOESM1_ESM.docx]

**Figure S1.** (A) The transfection efficiencies of shCHPF and shCtrl in A375 and OM431 cells were evaluated through detecting the fluorescence of green fluorescent protein tagged on the lentivirus vector (Magnification 200×).

**Figure S2.** (A) The difference in the expression of DEGs in shCtrl and shCHPF groups was shown by scattered plot. (B) Volcano plot shows the DEGs between the two groups. The red dots indicated the up-regulated ones, the blue dots indicated the down-regulated ones. (C) IPA analysis of canonical signaling pathway was performed to identify the pathways regulated by CHPF. The dots were scaled by –Log (p value). The pathways in red were activated, and these in blue were inhibited. (D) IPA analysis of disease and function was performed to explore the related disease or function regulated by CHPF knockdown. The dots were scaled by –Log (p value). (E) Several significantly up-regulated DEGs were selected for verification.

**Figure S3.** (A) The background expression of CHPF and CDK1 was detected by qPCR in A375 cell line. (B) Transfection efficiency of CHPF over-expression plasmids was evaluated through fluorescent microscopy as mentioned before (Magnification 200×). The over-expression of CHPF was verified by qPCR (C) and WB (D). (E) Transfection efficiency of CDK1 knockdown plasmids was evaluated through fluorescent microscopy as mentioned before (Magnification 200×). The over-expression of CHPF was verified by qPCR (F) and WB (G). The data were expressed as mean ± SD (n ≥ 3), **P*<0.05, ***P*<0.01, ****P*<0.001.
