# Supplementary figures and images for: Chondroitin polymerizing factor (CHPF) promotes development of malignant melanoma through regulation of CDK1
Source: Cell Death Dis. 2020 Jul 1;11(7):496. doi: 10.1038/s41419-020-2526-9 (PMC7329816; doi:10.1038/s41419-020-2526-9)

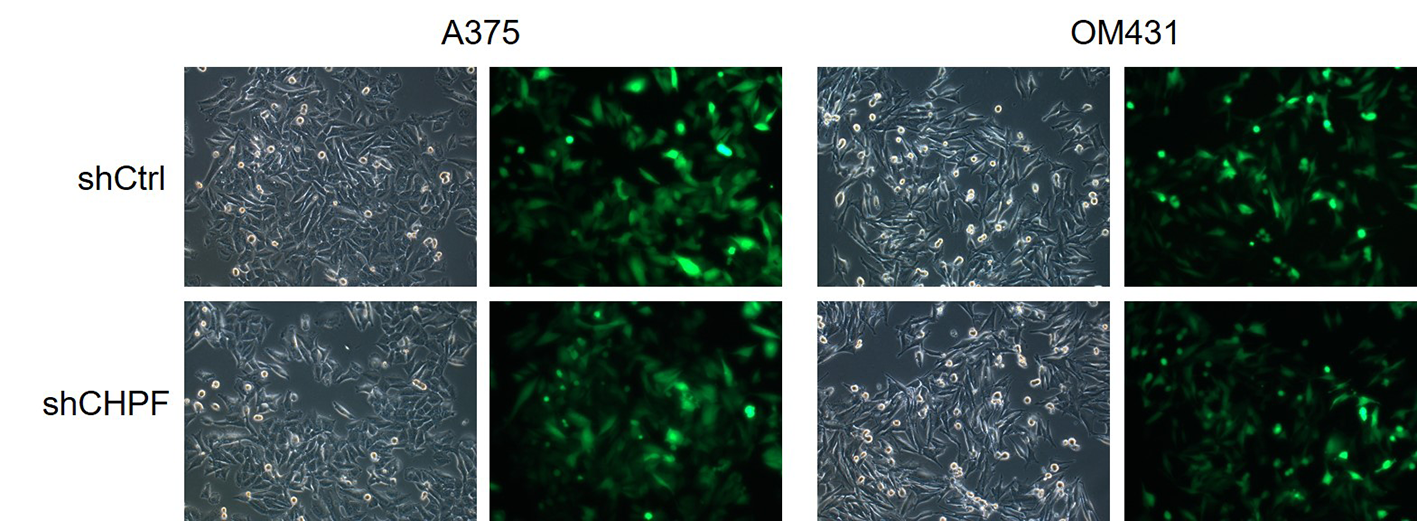

Supplement: Supplementary file 2 — Figure S1 [file 41419_2020_2526_MOESM2_ESM.tif]

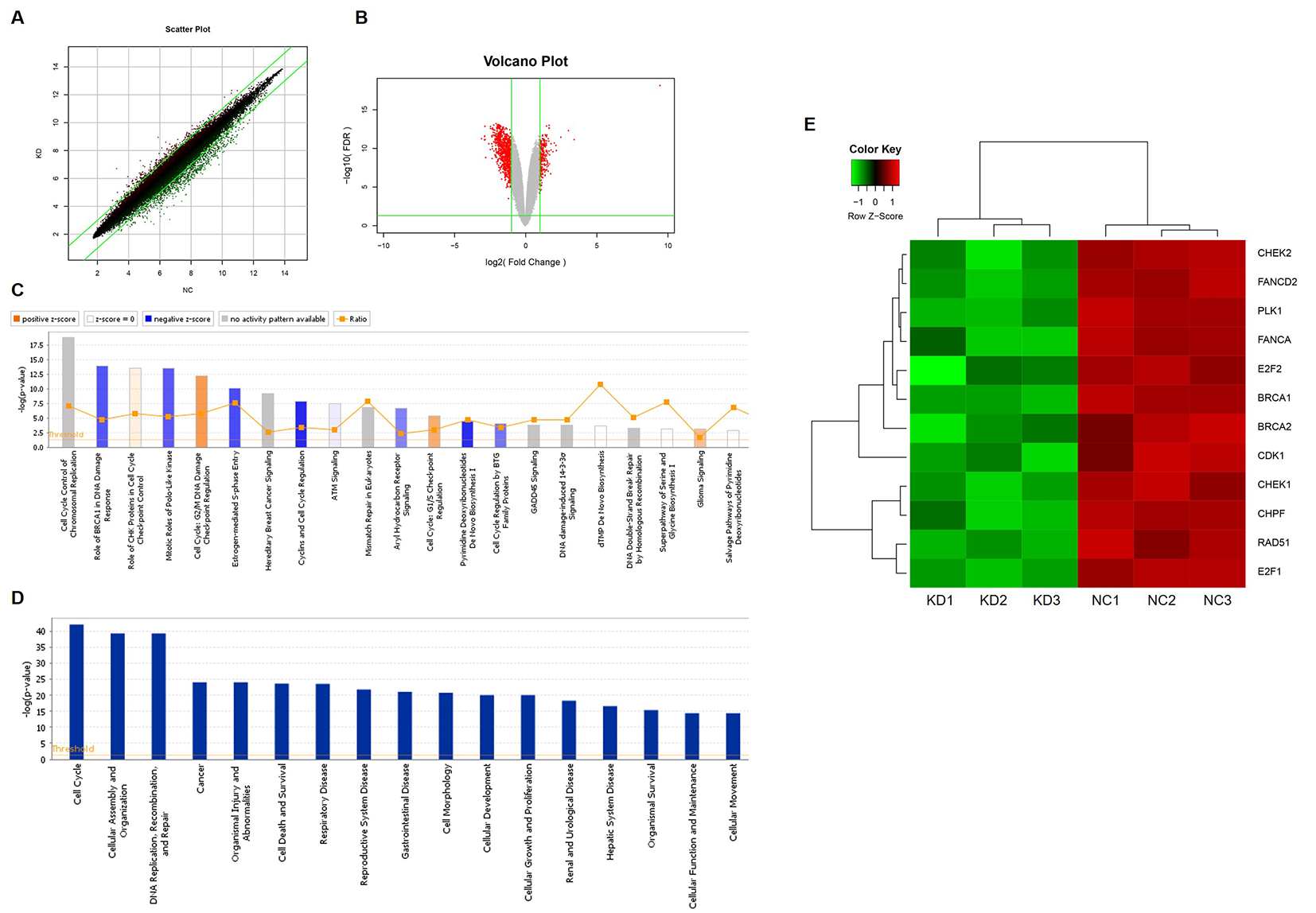

Supplement: Supplementary file 3 — Figure S2 [file 41419_2020_2526_MOESM3_ESM.tif]

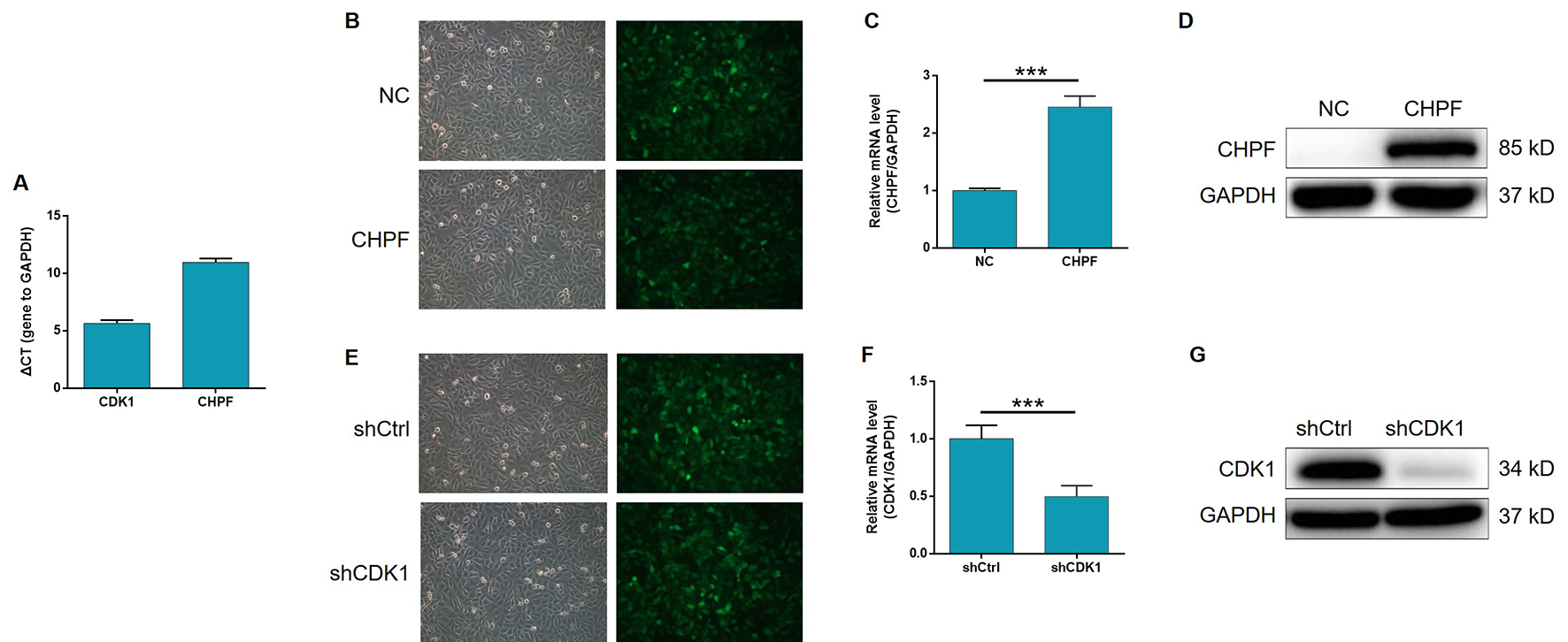

Supplement: Supplementary file 4 — Figure S3 [file 41419_2020_2526_MOESM4_ESM.tif]
